# Papillary renal neoplasm with reverse polarity: A case report

**DOI:** 10.3389/fonc.2023.1072213

**Published:** 2023-04-18

**Authors:** Chengjuan Xing, Hui Tian, Yunkun Zhang, Li Zhang, Jixia Kong

**Affiliations:** ^1^ Department of Pathology, The Second Affiliated Hospital of Dalian Medical University, Dalian, China; ^2^ Department of Emergency Medicine, Dalian Municipal Central Hospital Affiliated of Dalian University of Technology, Dalian, China

**Keywords:** papillary renal neoplasm with reverse polarity, indolent, GATA3, *KRAS*, case report

## Abstract

As a recently named rare renal tumor of epithelial origin, papillary renal neoplasm with reverse polarity (PRNRP) has unique histomorphological features and immunophenotypes, often associated with KRAS mutations and showing indolent biological behavior. In this study, we report a case of PRNRP. In this report, nearly all tumor cells were positive for GATA-3, KRT7, EMA, E-Cadherin, Ksp-Cadherin, 34βE12, and AMACR in varying intensities, focally positive for CD10 and Vimentin, while negative for CD117, TFE3, RCC, and CAIX. *KRAS* mutations (exon 2) were detected by amplification refractory mutation system polymerase chain reaction (ARMS-PCR), while no *NRAS* (exon 2-4) and *BRAF* V600 mutations (exon 15) were detected. A transperitoneal Robot-Assisted Laparoscopic Partial Nephrectomy was performed on the reported patient. No recurrence or metastasis was found during the 18 months of follow-up.

## Introduction

Papillary renal cell carcinoma (PRCC) was classified by Delahunt and Eble in 1997 into type 1 and type 2 as the second most common carcinoma of renal cells, depending on the tumor histomorphology and clinical prognosis (1, 2). Since 2003, a type of PRCC with favorable prognoses has been continuously reported, with eosinophilic granule-rich cytoplasm and non-overlapping low-grade nuclei. They are called oncocytic PRCC, which cannot be classified as traditional type 1 or type 2 (3–6). In 2019, Al-Obaidy et al. collected 421 cases of PRCC diagnosed at the pathology departments of three medical institutions from 2004 to 2017, from which they selected 18 morphologically similar tumors that differed from traditional PRCC tumors according to the morphological traits and analyzed immunophenotype and chromosomal of these tumors and found their indolent biological behavior. Thus, this tumor was named papillary renal neoplasm with reverse polarity (PRNRP) (7). Another study by Al-Obaidy et al. in the same year demonstrated that the PRNRP is associated with a high frequency of *KRAS* mutations (8).

Specifically, this tumor type is different from PRCC. PRCC type 1 tumors comprise papillary and tubular structures, where foamy histiocytes are aggregated in clusters inside the fibrovascular axis. The tumor cells are arranged in monolayers, with sparse basophilic or clear cytoplasm and low-grade nuclei. In PRCC type 2 tumors, histiocytes are rarely seen inside the fibrovascular axis, and tumor cells exhibit pseudostratified arrangement with abundant eosinophilic cytoplasm, high nuclear grade, and neoplastic necrosis. In contrast, PRNRP is composed primarily of papillary architectures, which can be accompanied by varying numbers of tubular-papillary structures. The tumor cells are arranged in monolayers with identical morphology, abundant eosinophilic cytoplasm, and low-grade nuclei. Their nuclei are located at the cytoplasmic top, far from the basement membranes, exhibiting reverse polarity. Immunophenotypically, PRCC tumors are often positive for AMACR, RCC, KRT7, and Vimentin but are negative for GATA3 and 34βE12, whereas PRNRP reveals diffuse staining of GATA3, L1CAM, and 34βE12 but is almost negative for Vimentin.

So far, only about 18 studies have been reported with the diagnostic term PRNRP, with a total of approximately 200 cases. Here, we report a case of PRNRP recently diagnosed in our department.

## Case report

A 57-year-old male presented with a mass in the right kidney on physical examination four years ago, who did not receive any therapy. In the past half month, the patient felt occasional pain in bilateral upper limbs, abdomen, and neck, but no low back pain or gross hematuria. Ultrasonography showed a bump in the middle of the right kidney, with slightly hyperechoic, a clear boundary, and no blood flow signal. Enhanced computed tomography of the lower abdomen showed quasi-circular isodense shadows of 2.9 cm × 2.5 cm in the right kidney, with arc-shaped calcifications at the edge and a CT value of 34 HU. The patient underwent A transperitoneal Robot-Assisted Laparoscopic Partial Nephrectomy.

Gross examination: a mass of 3 cm ×2.5 cm ×1.5 cm in size was observed on the cut surface of partial kidney tissue (size: 3.8 cm x 3 cm x 2 cm), with clear demarcation and intact capsule. The cut surface of the tumor was grayish-yellow, thin papillary, and brittle, which was tightly adjacent to the renal capsule.

**Figure 1:**
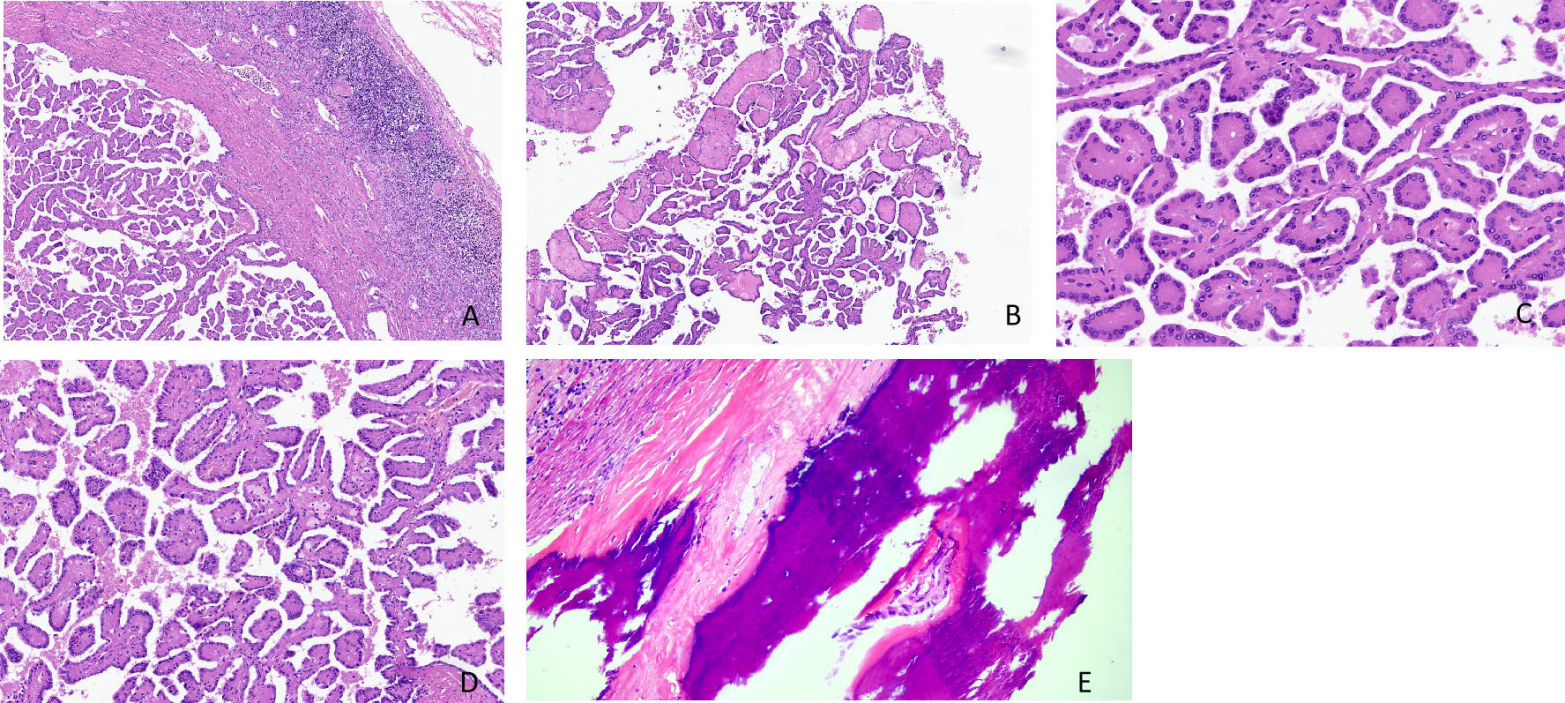
**(A–E)** Histomorphological features of papillary renal neoplasm with reverse polarity. The tumor is well defined, with a fibrous pseudocapsule and many lymphocyte infiltrates at the capsule margin (H&E stain, ×40) **(A)**. It showed a slender papillary structure with local tubular papillary branches (H&E stain, ×40) **(B)**. Low-grade nuclei, inverted linear nuclear location (H&E stain, ×200) **(C)**. A few foamy macrophages are observed in the stroma (H&E stain, ×100) **(D)**. Osseous metaplasia was locally visible in the tumor (H&E stain, ×200) **(E)**.

Microscopically, the tumor was clearly demarcated, with a fibrous pseudocapsule, and multiple lymphocytic infiltrates were present at the capsule edge (**Figure 1A**). The tumor cells were mostly in a slender papillary architecture, while locally in a tubular papillary shape. Papillary branching was visible, with a few large papillae and others edematous (**Figure 1B**). The tumor cells were morphologically uniform, cuboidal in shape, and arranged in monolayers, with abundant eosinophilic granules in the cytoplasm. Their nuclei were either round or oval, located at the cytoplasmic top and exhibiting a “reverse polar” arrangement. Their nucleoli were inconspicuous, corresponding to the WHO/ISUP grade 1, and no mitotic figures were noted (**Figure 1C**). Mast cells infiltrated the papillary stroma, and a few macrophages were focally observed, which phagocytosed some hemosiderin granules (**Figure 1D**). Focal papillary stroma underwent hyaline degeneration. Osseous metaplasia was locally visible in the tumor (**Figure 1E**). No tumor necrosis or psammoma body was observed.

**Figure 2:**
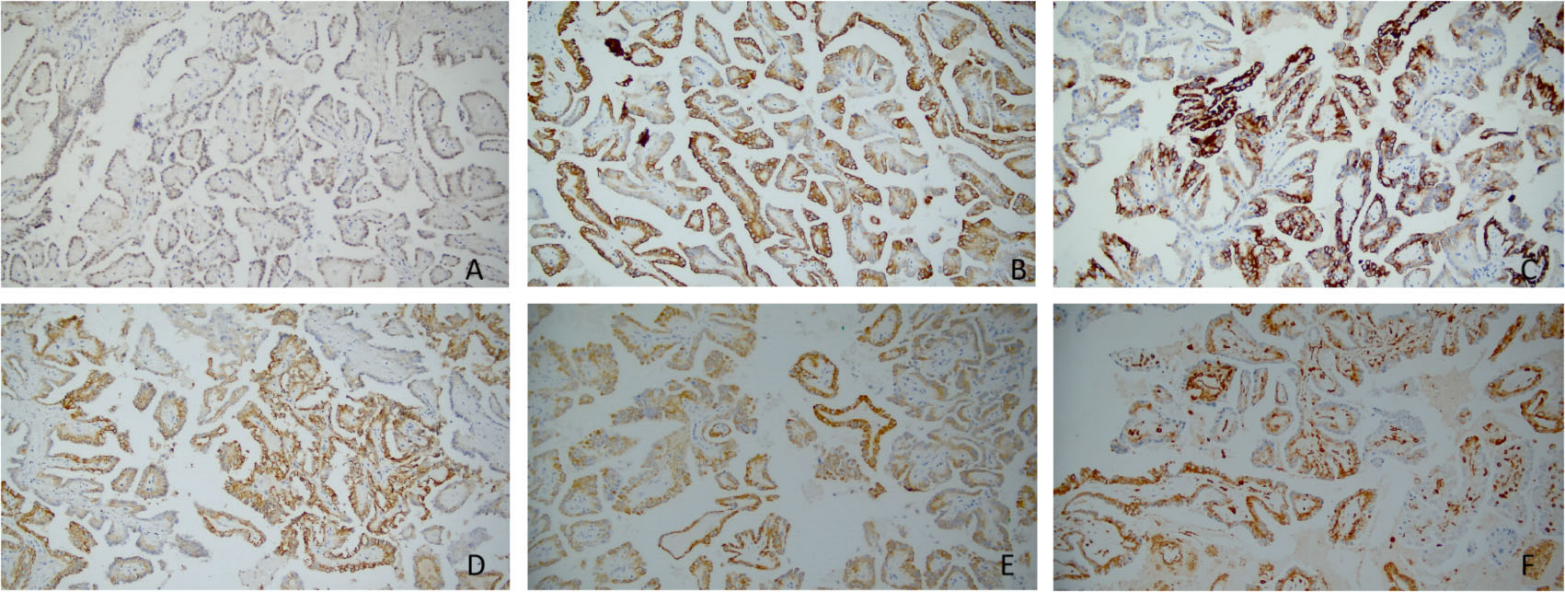
**(A–F)** Immunohistochemical profiles of papillary renal neoplasm with reverse polarity. The tumor cells are positive for GATA-3(×200) **(A)**, KRT7(×200) **(B)**, 34βE12(×200) **(C)**, Ksp-Cadherin(×200) **(D)**, AMACR(×200) **(E)**, and Vimentin(×200) **(F)**; however, the staining intensities varied.

Immunohistochemically, nearly all tumor cells expressed GATA-3, KRT7, EMA, 34βE12, E-cadherin, Ksp-Cadherin, and AMACR (**Figures 2A–E**). However, the staining intensities varied. Furthermore, the tumor cells were positive for CD10 and Vimentin at a few foci (**Figure 2F**), while negative for CD117, TFE3, RCC, and CAIX. The Ki-67 proliferation index was approximately 1%, and SDHB expression was not lost.

**Figure 3:**
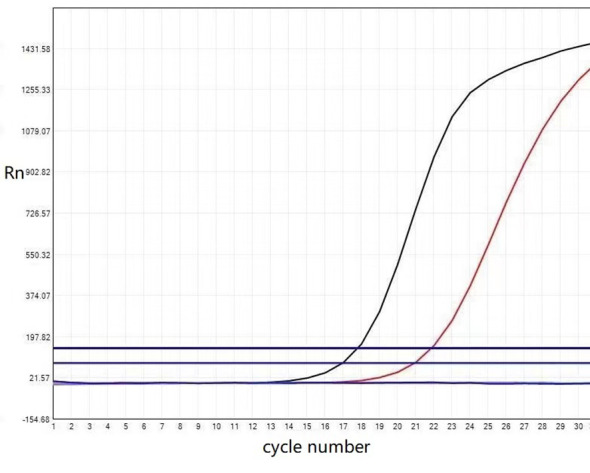
The KRAS mutation status of the papillary renal neoplasm with reverse polarity. Real-time fluorescent quantitative polymerase chain reaction analysis showed the KRAS mutation was present in exon 2-codon 12: p.G12V (c.35 G >T).

Real-time fluorescent quantitative polymerase chain reaction was used to detect the somatic hot spot mutation of Exon 2, 3, and 4 of *KRAS* and *NRAS* genes and the mutation of *BRAF* gene V600. The results showed the presence of *KRAS* Exon 2 mutation (**Figure 3**), while no *NRAS* or *BRAF* V600 mutation was detected. Finally, we made the pathological diagnosis of papillary renal neoplasm with reverse polarity (PRNRP) for the tumor.

## Discussion

Approximately 15% of renal cell carcinomas are PRCCs, whereas 1.3–9.1% of PRCCs are PRNRPs (1, 9, 10). PRNRP is more common among the middle-aged and elderly (46–84 years), without significant gender predilection. It often occurs in the right kidney, while rarely in both kidneys. PRNRPs are discovered accidentally during physical examination since most patients have no apparent symptoms, except for some with hematuria or low back pain (7, 10–12).

PRNRPs are 0.8–8 cm in diameter and well-demarcated with a fibrous pseudocapsule (7, 12). Tumor periphery lymphocyte infiltration can be seen microscopically. The tumor mainly comprises a slender papillary architecture with a fibrovascular axis and more or less tubular structures. Thick papillae or interstitial hyaline degeneration and cystically dilated papillae are observable. Papillary edema may appear, and small amounts of foamy histiocytes or hemosiderin-phagocytosing macrophages may be present in the papillary stroma. Papillae or canalicular lumens are smooth and covered with a monolayer of cuboidal tumor cells. Their cytoplasm contains abundant eosinophilic granules. Some tumor cells may have intracytoplasmic vacuoles, and clear cytoplasm is occasionally seen in focal tumors. Tumor cells have characteristic nuclei located at the cytoplasmic top far away from the basement membranes, exhibiting reverse polarity. The nuclei have uniform size and regular shape, which do not overlap. Occasionally, the nuclei are transparent, pyknotic, or mildly irregular, with inconspicuous nucleoli (WHO/ISUP grade 1–2). No psammoma bodies, mitotic figures, or tumor necrosis are present, and intratumoral osseous metaplasia is occasionally visible (7, 10, 12–16). The tumor histomorphology in the present study is consistent with previous reports.

A study by Al-Obaidy et al. showed that all tumor cells are diffusely and strongly positive for EMA and GATA3, moderately strongly positive for L1CAM, diffusely and strongly positive for MUC1 and AE1/AE3 (except for one case), and focally positive for CD10. Besides, most tumor cells are diffusely positive for KRT7 and reacting for AMACR to varying degrees but negative for Vimentin and CD117 (7). However, according to the study by Kim et al., most tumor cells are positive for KRT7 and EMA, and variably positive for AMACR and Vimentin, but negative for CD10 (13). It implies PRNRP could focally express CD10 and Vimentin. Zhou L et al. found that all 7 PRNRP cases expressed 34βE12 diffusely and strongly, whereas only 12 of 183 PRCC cases did. 34βE12 is, therefore, conducive to PRNRP/PRCC differentiation (12). A study by Kim et al. demonstrated that PRNRP could express E-cadherin in varying degrees, while Ji RH et al. reported that tumor cells expressed E-cadherin diffusely and strongly in all 9 cases of PRNRP. Thus, E-cadherin can also serve as an adjuvant diagnostic immunomarker for PRNRP (13, 17). In the PRNRP patient described in this report, nearly all tumor cells were positive for GATA-3, KRT7, EMA, E-Cadherin, Ksp-Cadherin, 34βE12, and AMACR in varying intensities, focally positive for CD10 and Vimentin, while negative for CD117, TFE3, RCC, and CAIX, showing agreement with previous reports.

According to recent research, PRNRP is often associated with *KRAS* mutations, primarily involving codon 12 in exon 2. A literature review reveals that 78 of 92 (84.8%) PRNRP cases harbor *KRAS* mutations (8, 10, 11, 13–19). In the present report, *KRAS* mutations (exon 2) were detected by amplification refractory mutation system polymerase chain reaction (ARMS-PCR), while no *NRAS* (exon 2-4) and *BRAF* V600 mutations (exon 15) were detected, showing a good agreement with the literature reports. Al-Obaidy et al. performed Fish chromosomal detection on 15 PRNRP cases, finding that 33% of the patients had trisomy 7, 33% had trisomy 17, and 20% had both trisomies 7 and 17. Besides, 14% of the male patients had Y chromosomal deletions without deleting 3p (7). Contrastively, in the studies of Al-Obaidy et al. and Ji RH et al., no abnormalities of chromosomes 7, 17, or Y were detected (16, 17). It is thus inferred that chromosomal abnormalities are less common in PRNRP than in PRCC, which also confirms that PRNRP resembles but differs from PRCC.

PRNRP should be differentiated from various renal tumors with papillary architecture based on histological and immunohistochemical analyses of these tumor types. Firstly, as previously mentioned, it should be differentiated from PRCC, which is classified into type 1 and type 2. Secondly, PRNRP shares similarities with clear cell PRCC (CCPRCC) regarding reverse polarity of nuclei and diffuse staining of GATA3, but the latter has clear cytoplasm, specific cup-shaped staining of CAIX, diffusely Cyclin D1-positive nuclei, no AMACR expression, and no *KRAS* mutations. In contrast, PRNRP shows some reactivity for AMACR but no expression of CAIX and is often associated with *KRAS* mutations. Thirdly, papillary adenomas of the kidney are defined as small, low-grade, and unencapsulated tumors with papillary or tubular architecture that measure no larger than 15 mm, harboring similar immunophenotypes and molecular alterations as PRCC, which can be differentiated from PRNRP by IHC staining.

Regarding clinical staging, the PRNRPs reported in the extant literature were all at stage T1, primarily treated by local nephrectomy and occasionally by radical nephrectomy. Postoperative recurrence or metastasis is not reported.

In conclusion, the mild tumor cell morphology, low Ki-67 proliferation index, lack of tumor necrosis and mitosis, and no tumor recurrence or metastasis during follow-up suggest that PRNRP is an indolent renal tumor with an immunophenotype distinct from other renal cell carcinomas and is often associated with *KRAS* gene mutations; a few may have trisomy of chromosome7, 17 and loss of Y. Given the limited number of cases currently available, further research with more cases is required in the future.

## Patient perspective

This study was approved by the Ethics Committee of the Second Affiliated Hospital of Dalian Medical University. It was performed per the guidelines of the Declaration of Helsinki (No. BBMCEC2012063). Written informed consent to participate in this study was provided by the participant’s legal guardian. Written informed consent was obtained from the patient’s legal guardian for publishing any potentially identifiable images or data in this article.

## Data availability statement

The original contributions presented in the study are included in the article/supplementary material. Further inquiries can be directed to the corresponding author.

## Ethics statement

The studies involving human participants were reviewed and approved by Ethics Committee of the Second Affiliated Hospital of Dalian Medical University. Written informed consent to participate in this study was provided by the participants’ legal guardian/next of kin.

## Author contributions

CX and HT: manuscript writing. YZ: molecular detection analysis. LZ: immunohistochemical staining analysis. JK: final manuscript revision. All authors contributed to the article and approved the submitted version.
